# Opioid Timeliness in the Emergency Department and Hospitalizations for Acute Sickle Cell Pain

**DOI:** 10.1001/jamapediatrics.2025.2967

**Published:** 2025-09-02

**Authors:** Ibrahim Gwarzo, Keli D. Coleman, Kenneth McKinley, Angela M. Ellison, Elizabeth R. Alpern, Jacqueline Corboy, Selena Hariharan, Irina Topoz, Morgan Wurtz, Blake Nielsen, Lawrence J. Cook, Claudia R. Morris, Amanda M. Brandow, Andrew D. Campbell, Robert I. Liem, Rachelle Nuss, Charles T. Quinn, Alexis A. Thompson, Anthony Villella, Allison A. King, Ana Baumann, Warren Frankenberger, David C. Brousseau

**Affiliations:** 1 Department of Pediatrics, Nemours Children’s Health, Wilmington, Delaware; 2 Sidney Kimmel Medical College at Thomas Jefferson University, Philadelphia, Pennsylvania; 3 Department of Pediatrics, Section of Emergency Medicine, Medical College of Wisconsin and the Children’s Research Institute of Children’s Wisconsin, Milwaukee; 4 Children’s National Medical Center, Washington, DC; 5 Department of Pediatrics, Children’s Hospital of Philadelphia, Philadelphia, Pennsylvania; 6 Department of Pediatrics, Ann & Robert H. Lurie Children’s Hospital, Northwestern University Feinberg School of Medicine, Chicago, Illinois; 7 University of Cincinnati College of Medicine, Cincinnati Children’s Hospital, Cincinnati, Ohio; 8 Department of Pediatrics, Section of Emergency Medicine, Children’s Hospital Colorado, Aurora; 9 Nationwide Children’s Hospital, Columbus, Ohio; 10 University of Utah, Salt Lake City; 11 Department of Pediatrics, Emory University School of Medicine and Children’s Healthcare of Atlanta, Georgia; 12 Department of Pediatrics, Section of Hematology/Oncology/Bone Marrow Transplantation, Medical College of Wisconsin and Children’s Research Institute of Children’s Wisconsin, Milwaukee; 13 Center for Cancer and Blood Disorders, Children’s National Hospital, Washington, DC; 14 Division of Hematology, Oncology & Stem Cell Transplant, Ann & Robert H. Lurie Children’s Hospital of Chicago, Chicago, Illinois; 15 Center for Cancer and Blood Disorders, University of Colorado Anschutz Medical Campus, Aurora; 16 Division of Hematology, Cincinnati Children’s Hospital Medical Center, Cincinnati, Ohio; 17 Pediatric Hematology/Oncology, Washington University School of Medicine, St Louis, Missouri; 18 Washington University School of Medicine, St Louis, Missouri

## Abstract

**Importance:**

Guidelines for the timely emergency department (ED) administration of opioids for acute sickle cell disease (SCD) pain lack clear evidence showing associations with reduced hospitalizations.

**Objective:**

To evaluate the associations between the timely administration of multiple opioid doses during uncomplicated SCD pain episodes in children and hospitalization.

**Design, Setting, and Participants:**

This multisite cross-sectional study used data from the Pediatric Emergency Care Applied Research Network Registry. Included were 12 pediatric EDs from children’s hospitals with comprehensive SCD centers across the US. ED visits by children younger than 19 years with uncomplicated SCD pain at any of the 12 sites between January 1, 2019, and December 31, 2021, were included. Primary diagnosis codes were used to identify visits for SCD pain; visits with any other SCD complication were excluded. Initial data analysis was conducted from April 2024 to April 2025, including revisions. After-revision analyses were done between May and June of 2025.

**Exposures:**

The 2 exposures of interest were as follows: (1) time from ED arrival to first opioid administration (dichotomized as ≤60 minutes of arrival vs >60 minutes from arrival) and (2) time interval between the first and the second opioid administration (dichotomized in separate analyses as ≤30 minutes vs >30 minutes, ≤45 minutes vs >45 minutes, and ≤60 minutes vs >60 minutes).

**Main Outcomes and Measures:**

The primary outcome was hospitalization at the end of an ED visit.

**Results:**

A total of 2538 patients (mean [SD] age, 12.0 [5.0] years; 1293 male [50.9%]) were included in this study. Of 9233 ED visits, 5023 (54.4%) resulted in hospitalization. First-dose timeliness of opioid administration evaluated alone was associated with decreased hospitalizations (odds ratio [OR], 0.84; 95% CI, 0.75-0.95). Evaluating combined first- and second-dose timeliness of opioid administration for the 7853 visits (85.1%) with 2 or more opioid doses, first-opioid timeliness was associated with reduced odds of hospitalization, even when the second dose was outside 30 minutes (OR, 0.85; 95% CI, 0.74-0.98) or 45 minutes (OR, 0.84; 95% CI, 0.72-0.97). First-dose timeliness combined with a second dose within 30-minute (OR, 0.62; 95% CI, 0.52-0.75), 45-minute (OR, 0.70; 95% CI, 0.59-0.83), and 60-minute (OR, 0.78; 95% CI, 0.67-0.92) intervals achieved the lowest odds of hospitalization.

**Conclusions and Relevance:**

This cross-sectional study found that timely receipt of opioids was associated with a reduction in hospitalizations for SCD pain. The largest decrease in hospitalization was achieved with the first dose within 60 minutes and the second dose within 30 minutes; however, longer second-dose durations were associated with reduced hospitalizations.

## Key Points

**Question:**

Is timely administration of opioids in the emergency department (ED) associated with hospitalization for sickle cell disease (SCD) pain?

**Findings:**

In this cross-sectional study of 2538 children younger than 19 years, for the majority of patients, 2 doses of opioids were administered during their visits to the ED. Receiving the first dose within an hour of ED arrival and the second dose within one-half hour of the first dose was associated with a reduction in the odds of hospitalization.

**Meaning:**

Results suggest that timely administration of both first and second opioid doses was associated with decreased hospitalizations in children presenting with SCD pain episodes.

## Teaser

This cross-sectional study investigates if timely administration of opioids in the emergency department is associated with hospitalization for sickle cell disease pain.

## Introduction

Sickle cell disease (SCD), a hemoglobinopathy disproportionately affecting Black and Latino populations^1,2^ affects 100 000 people in the US, with nearly 40% being children.^2,3^ Severe acute painful episodes, the hallmark of SCD, are the leading cause of emergency department (ED) visits.^4,5^ These painful episodes, if not able to be managed at home, require immediate medical attention.

Although some children with SCD pain present with other SCD complications, including acute chest syndrome or splenic sequestration, uncomplicated SCD pain (ie, would not require hospitalization if their pain were adequately controlled) accounts for the majority of ED presentations. Treating acute SCD pain in the ED requires timely evaluation and management using opioid-based medications.^6,7^

Guidelines from the American Society of Hematology (ASH) and the National Heart, Lung, and Blood Institute (NHLBI) provide recommendations for timely pain medication administration for acute SCD pain.^8,9^ Specifically, both the NHLBI and ASH recommend first opioid administration within 60 minutes of ED arrival.^9,10,11^ NHLBI guidelines recommend medication readministration every 30 minutes until pain is controlled.^9^ The ASH guideline differs slightly, recommending subsequent dosing every 30 to 60 minutes.^8^ Despite these recommendations, timely opioids administration for SCD pain in the ED is poor.^10,12^ Reasons for poor guideline adherence are multifaceted, including facility staffing shortages and room availability^13,14,15^; practitioner discomfort with the opioid dosages and frequency required to control pain; concerns about possible adverse effects; and implicit bias.^14,15^

Another significant barrier is the fact that the guideline relies on expert opinion for the recommendations rather than evidence showing that timeliness reduces hospitalization rates. Inconsistent results from single-site studies,^16,17^ compounded by the failure to adequately assess the combined impact of multiple opioid doses, lead to the uncertainty surrounding the impact of timely pain treatment. Additionally, recent work suggests the association between ED intranasal fentanyl use, treatment timeliness, and disposition needs further exploration.^18^

We addressed these evidence gaps using a multicenter study evaluating the timely administration of multiple opioid doses for uncomplicated SCD pain in children. We hypothesized that timely administration of the first and second opioid doses, at various guideline intervals, would be associated with decreased hospitalizations. We also explored intranasal fentanyl’s impact on timeliness and disposition.

## Methods

### Database, Study Design, and Cohort Identification

This was a cross-sectional analysis of data from the Pediatric Emergency Care Applied Registry Network (PECARN) Registry. The PECARN Registry is a data repository of clinical information from patients’ electronic health records during ED encounters across multiple pediatric ED sites in the US. The Registry includes demographic information, diagnosis codes, ED arrival times, ED disposition, pain scores, and medication types, routes, and administration times. PECARN’s origin and ongoing work were described elsewhere.^19,20^ This study was exempt from obtaining individual participant consent by each site’s institutional review boards due to prior approval from a registry. Findings were reported according to the Strengthening the Reporting of Observational Studies in Epidemiology (STROBE) reporting guidelines.

We included data from all 12 EDs that participate in the registry including Ann & Robert H. Lurie Children’s Hospital, Children’s Healthcare of Atlanta at Egleston, Children’s Healthcare of Atlanta at Hughes Spalding, Children’s Healthcare of Atlanta at Scottish Rite, Children’s Hospital Colorado, Children’s Hospital of Philadelphia, Children’s National Medical Center, Children’s Wisconsin, Cincinnati Children’s Hospital, Nationwide Children’s Hospital, UC Davis Medical Center, and University of Michigan C.S. Mott Children’s Hospital. We used a validated algorithm with more than 90% accuracy in identifying children with acute uncomplicated SCD pain, to identify the eligible childhood visits from electronic health record data within the PECARN Registry.^10^

### Population Definition

The study included children presenting between January 1, 2019, and December 31, 2021, and meeting the following criteria: age younger than 19 years AND either primary *International Statistical Classification of Diseases and Related Health Problems, Tenth Revision *(*ICD-10*) diagnosis code for SCD (*ICD-10* = D57* except sickle cell trait) or presenting chief complaint of sickle cell pain or fever AND receipt of at least 1 parenteral opioid. Patients with a complicated pain event, as defined by an *ICD-10* diagnosis code of acute chest syndrome, priapism, splenic sequestration, or documented temperature in the ED greater than 38.5 °C, were excluded. The unit of analysis was ED visits; patients could have multiple visits over the study period. Preliminary evaluation revealed that across 70% eligible visits, both the first and second opioid doses were parenteral; we performed a secondary analysis for visits where the first 2 opioid doses were parenteral. Opioids administered through any route other than the oral route were considered parenteral.

Information on participant race and ethnicity was gathered from the EHR on the participants; however, these data were not used in the analysis. Participant race and ethnicities were identified as non-Hispanic Black, Hispanic, non-Hispanic White, or non-Hispanic other, which included Asian and Native.

### Measures

This primary outcome was hospitalization at the end of an ED visit. We used ED disposition from the registry to categorize patients as hospitalized vs discharged. We excluded visits with dispositions of left against medical advice and left without being seen, transferred, or as a result of death (combined <5% excluded). The exposure of interest was the opioid administration timing, operationalized as 2 distinct variables: (1) time from ED arrival to first opioid administration (dichotomized as ≤60 minutes vs >60 minutes from arrival) and (2) time between the first and the second opioid administration (dichotomized in separate analyses consistent with NHLBI and ASH guidelines as ≤30 minutes vs >30 minutes, ≤45 minutes vs >45 minutes, and ≤60 minutes vs >60 minutes). Visits with fewer than 2 opioid doses were excluded from the analyses evaluating second-dose timeliness and hospitalization. We adjusted for potential confounders, including age (<12 years vs 12-19 years), sex (male vs female), site, and first pain score at presentation (range, 0-10).

Although a third dose was administered in 58% of visits, analysis of third-dose timeliness was not reported due to small numbers of timely administered doses, especially when combined with first- and second-dose timeliness.

### Statistical Analysis

We used χ^2^ and *t* tests to compare visit-level demographics, summarizing the disposition rates according to the sites and demographic variables. A multivariable logistic regression model evaluated the association between the first opioid dose timeliness and hospitalization, controlling for demographic variables, site differences, and the initial ED pain score. We applied generalized estimating equations and an independent correlation structure to account for within-patient clustering. We did not adjust for intravenous ketorolac administration, as preliminary findings indicated a link between ED ketorolac use and hospitalization; however, this association disappeared when limited to cases where ketorolac was given within 30 minutes of the first opioid dose, suggesting that its use often occurred after an inadequate response to opioids.

For visits with multiple opioid doses, the individual and joint associations of timely opioid administration with hospitalization were explored by categorizing ED visits into 4 distinct groups based on timeliness: (1) first dose administered greater than 60 minutes after arrival and the second dose greater than 30 minutes after the first (reference), (2) first dose administered 60 minutes or less after arrival and the second dose greater than 30 minutes after the first, (3) first dose administered greater than 60 minutes after arrival and the second dose 30 minutes or less after the first, and (4) first dose administered 60 minutes or less after arrival and the second dose 30 minutes or less after the first. We used similar categorizations to evaluate the second-dose associations within 45 and 60 minutes, respectively.

Because the impact of opioid timing on hospitalization may vary according to the patient’s initial pain severity, we examined the interaction between the initial ED pain score and the timely opioid administration. There was a statistically significant interaction between receipt of the first opioid within 60 minutes and the first ED pain score; however, this interaction only applied to 5% of visits with first ED pain scores of 2 or less and was therefore deleted. Additionally, due to the potential for a residual unmeasured confounder in our study, we used a previously reported methodology^21^ to calculate the E-values estimating the strength of the association for a potential residual confounder to reduce the estimated association we report.

A priori knowledge and the literature suggested a link between intranasal fentanyl and first opioid dose^18^ timeliness. Our data showed greater than 90% of ED visits that received intranasal fentanyl had the first opioid dose within 60 minutes of arrival, indicating the 2 factors are highly correlated (phi correlation coefficient *r*_φ_ = 0.48). Therefore, intranasal fentanyl was not adjusted for in the main multivariable analysis, but in a supplementary analysis, we stratified the adjusted odds ratios (ORs) according to receipt of intranasal fentanyl to disentangle the association between the first opioid dose timeliness and intranasal fentanyl.

The COVID-19 pandemic started within our study’s duration, potentially impacting hospitalizations across the sites; however, our comparison revealed no difference in overall hospitalizations before March 1, 2020, and after March 1, 2020.

Statistical analyses were conducted using SAS/STAT software, version 9.4 (SAS Institute). All 95% CIs, not including the null value, were interpreted to infer statistically significant differences in the odds of hospitalization. All *P* values were 2-sided, and *P* <.05 was considered statistically significant. Initial data analysis was conducted from April 2024 to April 2025, including revisions. After-revision analyses were done between May and June of 2025.

## Results

Within the study period, the PECARN Registry had 10 570 ED visits for acute SCD pain. After exclusion criteria were applied, the final sample included 9233 ED visits from 2538 unique patients (Figure). All visits, by definition, had at least 1 opioid dose administered; 7853 (85.1%) received a second opioid dose, and 5364 (58.1%) received a third dose. Despite the start of the COVID-19 pandemic during our study, our comparison revealed no difference in overall hospitalizations before March 1, 2020 (OR, 54.2%; 95% CI, 52.7%-55.7%), and after March 1, 2020 (OR, 54.6%; 95% CI, 53.2%-56.0%).

**Figure.**
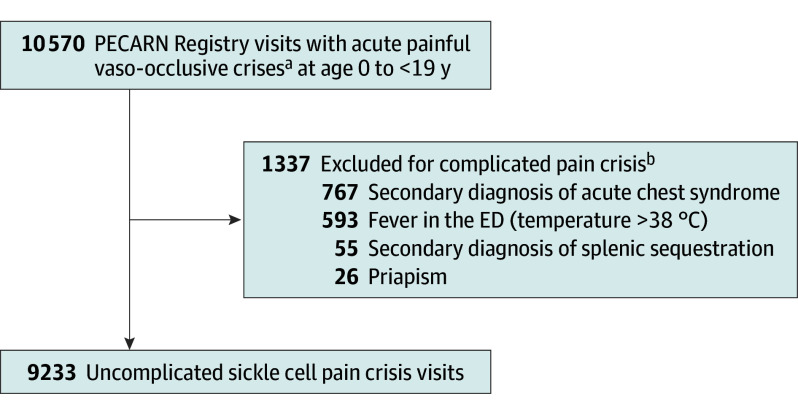
Study Flow Diagram PECARN indicates Pediatric Emergency Care Applied Registry Network. ^a^Primary *International Statistical Classification of Diseases and Related Health Problems, Tenth Revision *(*ICD-10*) diagnosis code for sickle cell disease, *ICD-10* = D57, except sickle cell trait OR a chief complaint of sickle cell pain crisis OR sickle cell fever AND received at least 1 parenteral opioid pain medication in the emergency department (ED). Visits from January 2019 through December 2021. ^b^Visits could be ineligible due to more than 1 exclusion criterion.

Table 1 summarizes visit-level demographic patient characteristics by disposition. A total of 2538 children (mean [SD] age, 12.0 [5.0] years; 1245 female [49.1%]; 1293 male [50.9%]) were included in this study. Of the 2538 participants, 2395 (94.8%) were non-Hispanic Black, 81 were Hispanic (3.2%), 5 were non-Hispanic White (0.2%), and 46 were non-Hispanic other race (1.8%). Admission variability across the 12 sites existed; the hospitalization rate ranged from 30% to 80%. Of 9233 ED visits, 5023 (54.4%) for acute uncomplicated SCD pain resulted in hospitalization. Visits by females and children 12 years and older were more likely to result in hospitalization.

**Table 1.** Emergency Department (ED) Visit Demographics by Hospital Admission

Demographic	Admitted, No. (%)		Overall (n = 9233),^a^ No.	*P* value
	No (n = 4210)	Yes (n = 5023)		
Site				
1	103 (29.0)	252 (71.0)	355	<.001^b^
2	251 (44.7)	310 (55.3)	561	
3	535 (53.8)	459 (46.2)	994	
4	569 (66.2)	290 (33.8)	859	
5	728 (41.8)	1012 (58.2)	1740	
6	42 (70.0)	18 (30.0)	60	
7	130 (40.5)	191 (59.5)	321	
8	727 (42.3)	991 (57.7)	1718	
9	20 (20.6)	77 (79.4)	97	
10	363 (35.5)	660 (64.5)	1023	
11	700 (50.3)	692 (49.7)	1392	
12	42 (37.2)	71 (62.8)	113	
Age category, y				
<12	1968 (57.8)	1435 (42.2)	3403	<.001^b^
12-19	2242 (38.5)	3588 (61.5)	5830	
Sex				
Female	2119 (44.3)	2669 (55.7)	4788	.007^b^
Male	2091 (47.0)	2354 (53.0)	4445	
First ED pain score (range, 0-10)				
No.	4098	4989	9087	NA
Mean (SD)	7.1 (2.67)	8.2 (2.14)	7.7 (2.45)	NA
Median (IQR)	8.0 (6.0-9.0)	9.0 (7.0-10.0)	8.0 (7.0-10.0)	<.001^c^
First opioid within 60 min of ED arrival				
No	1275 (42.7)	1708 (57.3)	2983	<.001^b^
Yes	2935 (47.0)	3315 (53.0)	6250	
**Timeliness of 2 opioid doses administered** ^a^				
First >60 min and second >30 min	733 (35.0)	1362 (65.0)	2095	<.001^b^
First ≤60 min and second >30 min	1376 (39.3)	2124 (60.7)	3500	
First >60 min and second ≤30 min	71 (41.8)	99 (58.2)	170	
First ≤60 min and second ≤30 min	1028 (49.2)	1060 (50.8)	2088	
**Timeliness of 2 opioid doses administered** ^a^				
First >60 min and second >45 min	616 (35.0)	1146 (65.0)	1762	<.001^b^
First ≤60 min and second >45 min	1037 (39.6)	1583 (60.4)	2620	
First >60 min and second ≤45 min	188 (37.4)	315 (62.6)	503	
First ≤60 min and second ≤45 min	1367 (46.1)	1601 (53.9)	2968	
**Timeliness of 2 opioid doses administered** ^a^				
First >60 min and second >60 min	502 (37.0)	854 (63.0)	1356	<.001^b^
First ≤60 min and second >60 min	752 (39.8)	1137 (60.2)	1889	
First >60 min and second ≤60 min	302 (33.2)	607 (66.8)	909	
First ≤60 min and second ≤60 min	1652 (44.7)	2047 (55.3)	3699	

Abbreviation: NA, not applicable.

^a^ N = 7853 for visits where a second opioid dose was received.

^b^ Result of χ^2^ test.

^c^ Result of *t* test.

### Timeliness of First Opioid Only and Hospitalization

Overall, 3315 of 5023 visits (53.0%) resulted in hospitalization when the first opioid was administered within 60 minutes vs 1708 of 5023 (57.3%) when the first opioid was administered after 60 minutes, a 4.2% (95% CI, 2.1%-6.4%) decrease. The multivariable analysis shows that receiving the first opioid within 60 minutes of ED arrival was associated with decreased odds of hospital admission across the 9087 visits included in the model (OR, 0.84; 95% CI, 0.75-0.95).

### Timeliness of First and Second Opioids and Hospitalization

Table 2 shows the regression results evaluating the association between having the first opioid dose within 60 minutes and having, or not having, the second dose within a 30-, 45-, and 60-minute interval for the 7853 visits (85.1% of all visits) with at least 2 opioid doses. Overall, improved opioid administration timeliness was associated with decreased odds of hospitalization.

**Table 2.** Associations Between Hospital Admission and Guideline Adherent Opioid Administration for Multiple Doses of Opioids—Visits With at Least 1 Parenteral Opioid (N = 7753)

Demographic	Sample size, No. (%)	Hospitalization rate, %	Adjusted OR (95% CI)		
			Model 1^a^	Model 2^a^	Model 3^a^
First opioid ≤60 min of ED arrival					
No	2951 (32.5)	57.3	NA^b^	NA	NA
Yes	6136 (67.5)	53.0	NA^b^	NA^b^	NA^b^
**Timeliness of first 2 opioid doses administered**					
First >60 min and second >30 min	2080 (26.8)	65.0	1 [Reference]		
First ≤60 min and second >30 min	3456 (44.6)	60.7	0.85 (0.74-0.98)	NA^b^	NA^b^
First >60 min and second ≤30 min	168 (2.2)	58.2	0.75 (0.54-1.04)	NA^b^	NA^b^
First ≤60 min and second ≤30 min	2049 (26.4)	50.8	0.62 (0.52-0.75)	NA^b^	NA^b^
**Timeliness of first 2 opioid doses administered**					
First >60 min and second >45 min	1751 (22.6)	65.0	NA^b^	1 [Reference]	NA
First ≤60 min and second >45 min	2590 (33.4)	60.4	NA^b^	0.84 (0.72-0.97)	NA^b^
First >60 min and second ≤45 min	497 (6.4)	62.6	NA^b^	0.82 (0.67-1.02)	NA^b^
First ≤60 min and second ≤45 min	2915 (37.6)	53.9	NA^b^	0.70 (0.59-0.83)	NA^b^
**Timeliness of first 2 opioid doses administered**					
First >60 min and second >60 min	1347 (17.4)	63.0	NA^b^	NA^b^	1 [Reference]
First ≤60 min and second >60 min	1866 (24.1)	60.2	NA^b^	NA^b^	0.92 (0.78-1.08)
First >60 min and second ≤60 min	901 (11.6)	66.8	NA^b^	NA^b^	1.08 (0.89-1.31)
First ≤60 min and second ≤60 min	3639 (46.9)	55.3	NA^b^	NA^b^	0.78 (0.67-0.92)

Abbreviations: ED, emergency department; NA, not applicable; OR, odds ratio.

^a^ Adjusted for age, sex, sites, and first ED pain scores.

^b^ Variable not included in the model.

#### Evaluating First-Dose Timeliness Without Second-Dose Timeliness

Lower odds of hospitalization were seen when the first opioid was received within 60 minutes and the second dose was not received within 30 minutes (OR, 0.85; 95% CI, 0.74-0.98) or not received within 45 minutes (OR, 0.84; 95% CI, 0.72-0.97). The association between the first-dose timeliness and hospitalization was no longer statistically significant when the second dose was not administered within 60 minutes of the first ( OR, 0.92; 95% CI, 0.78-1.08).

#### Evaluating Second-Dose Timeliness Without First-Dose Timeliness

The point estimates in second-dose timeliness, when the first dose was not received within 60 minutes, suggested a decrease in odds of hospitalization at both 30 minutes (OR, 0.75; 95% CI, 0.54-1.04) and 45 minutes (OR, 0.82; 95% CI, 0.67-1.02); however, the ORs were not statistically significant due to the sample sizes of only 168 and 497 in the 2 groups, respectively. There was no association between the second-dose timeliness and hospitalization when the second dose was given within 60 minutes (OR, 1.08; 95% CI, 0.89-1.31) of the first.

#### Combined First- and Second-Dose Timeliness

Receiving timely first and second doses of opioids was associated with a decrease in the odds of hospitalization. This association held regardless of the second dose’s timeliness: 30, 45, or 60 minutes of the first dose. Maximum reductions in the odds of hospitalization occurred when the interval between the first and the second dose was shorter, ie, the second dose within 30 minutes (OR, 0.62; 95% CI, 0.52-0.75) vs 45 minutes (OR, 0.70; 95% CI, 0.59-0.83) and 60 minutes (OR, 0.78; 95% CI, 0.67-0.92) of the first. There was no consistent statistically significant interaction between the timeliness of the first 2 opioids and the first ED pain scores across all pain score levels (range, 0-10).

The E-values for potential unmeasured confounders ranged from 1.25 to 1.56 across the opioid timeliness categories (exposures) (eTable in Supplement 1). This indicates that a residual confounder could explain the reported association if they had a relative risk association of 1.25 (lowest exposure category) or 1.56 (highest exposure category) with both second opioid timeliness and hospitalization.

#### Intranasal Fentanyl Analysis

The stratification by receipt of intranasal fentanyl demonstrates the small percentage of visits (256 of 3739 [6.9%]) with intranasal fentanyl administration and first opioid dose receipt greater than 60 minutes. For visits without intranasal fentanyl, first opioid dose timeliness was associated with lower odds of hospitalization whether the second was received within 30 minutes (OR, 0.73; 95% CI, 0.54-0.99) or not received within 30 minutes (OR, 0.84; 95% CI, 0.72-0.97) of the first dose (Table 3). For visits with intranasal fentanyl, a similar direction in point estimates for associations with timeliness was seen as visits without intranasal fentanyl but were not significant potentially due to the small number of referent group visits.

**Table 3.** Associations Between Hospital Admission and Guideline Adherent Opioid Administration for Multiple Doses of Opioids Stratified by Receipt of Intranasal Fentanyl

Demographic	Sample size, No. (%)	Visits without intranasal fentanyl (n = 4084), adjusted OR (95% CI)^a^	Sample size, No. (%)	Visits with intranasal fentanyl (n = 3669), adjusted OR (95% CI)^a^
**Timeliness of first 2 opioid doses administered**				
First >60 min and second >30 min	1921 (47.0)	1 [Reference]	159 (4.3)	1 [Reference]
First ≤60 min and second >30 min	1890 (46.3)	0.84 (0.72-0.97)	1566 (42.7)	0.95 (0.61-1.47)
First >60 min and second ≤30 min	74 (1.8)	0.72 (0.46-1.11)	94 (2.6)	0.87 (0.48-1.55)
First ≤60 min and second ≤30 min	199 (4.9)	0.73 (0.54-0.99)	1850 (50.4)	0.70 (0.45-1.08)

Abbreviation: OR, odds ratio.

^a^ Adjusted for age, sex, sites, and initial emergency department pain scores.

#### Secondary Analysis for the Exclusive Parenteral Opioid Visits

Significantly lower odds of hospitalization were noticed with the first dose within 60 minutes and/or the second dose within 30 or 45 minutes, except for the smallest group. For the second dose within 60 minutes, only combined dose timeliness was associated with lower hospitalizations (Table 4). These findings are consistent with the primary analysis.

**Table 4.** Associations Between Hospital Admission and Guideline Adherent Opioid Administration for Multiple Doses of Opioids—Visits Where the First and the Second Doses Were Parenteral (N = 5383)

Demographic	Sample size, No. (%)	Hospitalization rate, %	Adjusted OR (95% CI)		
			Model 1^a^	Model 2^a^	Model 3^a^
First opioid ≤60 min of ED arrival					
No	2805 (36.3)	57.1	NA^b^	NA	NA
Yes	4918 (63.7)	52.9	NA^b^	NA^b^	NA^b^
**Timeliness of first 2 opioid doses administered**					
First >60 min and second >30 min	1860 (34.6)	65.2	1 [Reference]	NA	NA
First ≤60 min and second >30 min	2602 (48.3)	61.3	0.85 (0.73-0.99)	NA^b^	NA^b^
First >60 min and second ≤30 min	105 (2.0)	57.3	0.69 (0.47-1.04)	NA^b^	NA^b^
First ≤60 min and second ≤30 min	816 (15.2)	50.8	0.66 (0.53-0.82)	NA^b^	NA^b^
**Timeliness of first 2 opioid doses administered**					
First >60 min and second >45 min	1539 (28.6)	65.3	NA^b^	1 [Reference]	NA
First ≤60 min and second >45 min	1888 (35.1)	61.0	NA^b^	0.82 (0.69-0.97)	NA^b^
First >60 min and second ≤45 min	426 (7.9)	62.6	NA^b^	0.76 (0.60-0.96)	NA^b^
First ≤60 min and second ≤45 min	1530 (28.4)	54.5	NA^b^	0.74 (0.61-0.89)	NA^b^
**Timeliness of first 2 opioid doses administered**					
First >60 min and second >60 min	1154 (21.4)	63.2	NA^b^	NA^b^	1 [Reference]
First ≤60 min and second >60 min	1320 (24.5)	60.3	NA^b^	NA^b^	0.87 (0.72-1.05)
First >60 min and second ≤60 min	811 (15.1)	67.1	NA^b^	NA^b^	0.97 (0.79-1.21)
First ≤60 min and second ≤60 min	2098 (39.0)	56.2	NA^b^	NA^b^	0.79 (0.66-0.95)

Abbreviations: ED, emergency department; NA, not applicable; OR, odds ratio.

^a^ Adjusted for age, sex, sites, and first ED pain scores.

^b^ Variable not included in the model.

## Discussion

This multicenter study demonstrates that timely opioid medication administration was associated with a significant reduction in hospitalization rates in children experiencing uncomplicated SCD pain. Specifically, we found that receiving the first opioid dose within 60 minutes was associated with decreased hospitalization across all children with uncomplicated SCD pain. Evaluating both first- and second-dose timeliness revealed a strong association between timeliness and hospitalization. When the first dose was administered within 60 minutes of arrival, as both the NHLBI and ASH guidelines recommend, the odds of hospitalizations were lower by 15%. Receipt of a second opioid dose within 30 and 45 minutes of the first dose was associated with a decrease in the odds of hospitalizations even when the first dose was delayed beyond 60 minutes, although the association was not statistically significant likely due to low sample size. Our analysis showed that when the first opioid dose was administered within 60 minutes of arrival, timely second dose administration within 30, 45, or 60 minutes was associated with the largest reduction in odds of hospitalizations. These results were consistent across visits with exclusive parenteral opioids and visits where both parenteral and oral opioids were administered.

This was the largest multicenter study, to our knowledge, that evaluated the association between the timely acute SCD pain management and ED disposition, filling an important gap by analyzing the individual and combined associations between multiple opioid doses and hospitalization. Two previous single-site studies^16,17^ showed no association between receipt of the first opioid within 60 minutes and lower odds of hospitalization. One of those studies^16^ found reduced odds of hospitalization with a second dose within 30 minutes; the other study^17^ did not evaluate second-dose timeliness. Neither study evaluated combined first- and second-dose timeliness.

A multisite study^18^ involving 400 patients found no association between receiving the first opioid within 60 minutes of ED arrival and odds of discharge; however, intranasal fentanyl was linked to a 9-fold decrease in hospitalization odds. The study did not account for potential collinearity between intranasal fentanyl administration and first-dose timeliness. In our analysis, the inclusion of both factors in the same regression model compromised the assumptions of independence. Our stratification indicated that timely opioid administration was associated with reduced hospitalization odds in children not receiving intranasal fentanyl. Differences in odds of hospitalization among those who received intranasal fentanyl suggest possible unmeasured facility-specific factors, warranting further investigation.

Our study addresses previous gaps by showing the overall association between timely opioid administration and hospitalization when analyzing first-dose timeliness, second-dose timeliness, and combined first- and second-dose timeliness. In addition, our analyses suggest lower odds of hospitalization when the second opioid dose is received within 45 minutes of the first in addition to 30 minutes. Given the difficulty in improving the ED medication administration timeliness, this finding is vital. ED teams should continue striving for improved timely administration of subsequent opioids even if not adherent to NHLBI guidelines, as improved timeliness could still decrease hospitalizations.

The NHLBI and ASH guidelines for acute SCD pain management in the ED relied on expert opinion due to a paucity of evidence.^8,9^ However, evidence from this study illuminates the disposition benefits of timely opioid administration. Reducing avoidable hospitalizations for uncomplicated SCD pain episodes indicates better pain control, improves resource availability, decreases overcrowding, and prevents other adverse outcomes associated with hospitalizations.^22,23^ Barriers such as the staffing and ED rooms, concern about opioid overuse among the SCD population, and implicit bias are hampering efforts to improve the timely management of SCD pain in the ED. Our findings of strong associations between timely opioid administration and lower odds of hospitalizations provide evidence to support implementing strategies to improve outcomes in children with SCD.

### Limitations

This study has several limitations. The data are retrospective and rely on documentation within the electronic health record; however, the PECARN Registry undergoes extensive data validation and has been widely used for evaluating quality of care metrics.^24,25,26,27,28^ Our study was restricted to children’s hospital sites participating in the PECARN Registry and may not be generalizable to children’s hospitals not participating in the registry. The population also does not include adults; data regarding opioid timeliness in general EDs is not readily available and may have a different association with disposition. Additionally, data regarding the care of patients before ED presentations are unavailable. However, this study does provide a comprehensive evaluation of first and second-dose timing for children with SCD presenting to the ED in acute pain.

## Conclusions

This cross-sectional study found that timely opioid administration according to the NHLBI and ASH recommended guidelines was associated with significantly decreased odds of hospitalization among children presenting to the ED with acute uncomplicated SCD pain. Although benefits were received when the first dose was administered within 60 minutes of ED arrival and when the second dose was administered within 30 or 45 minutes, the maximum reduction in the odds of hospitalizations was achieved when both the first and second doses were received in a timely manner.

## Notes

Supplement 1. **eTable. **Calculated E-Values for Timeliness of First 2 Opioid Doses Administered

Supplement 2.
Data Sharing Statement.
