# Supplementary material for: Opioid Timeliness in the Emergency Department and Hospitalizations for Acute Sickle Cell Pain
Source: JAMA Pediatr. 2025 Sep 2;179(11):1194–202. doi: 10.1001/jamapediatrics.2025.2967 (PMC12406144; doi:10.1001/jamapediatrics.2025.2967)
Supplement: Supplement 1. — eTable. Calculated E-Values for Timeliness of First 2 Opioid Doses Administered [file jamapediatr-e252967-s001.pdf]

Supplementary Online Content

Gwarzo I, Coleman KD, McKinley K, et al. Opioid timeliness in the emergency department and hospitalizations for acute sickle cell pain. *JAMA Pediatr*. Published online September 2, 2025. doi:10.1001/jamapediatrics.2025.2967

eTable. Calculated E-Values for Timeliness of First 2 Opioid Doses Administered

This supplementary material has been provided by the authors to give readers additional information about their work.

© 2025 Gwarzo I et al. *JAMA Pediatrics*.

eTable. Calculated E-Values for Timeliness of First 2 Opioid Doses Administered

Opioid Timeliness Category (Exposure)	Effect Size (OR)[1]	Effect Size (RR)[2]	E-value
**First >60 mins & second >30 mins**		**Reference**	
First ≤60 mins & second >30 mins	0.85[3]	0.95	1.29
First >60 mins & second ≤30 mins	0.75	0.99	1.11
First ≤60 mins & second ≤30 mins	0.62[3]	0.87	1.56
**First >60 mins & second >45 mins**		**Reference**	
First ≤60 mins & second >45 mins	0.84[3]	0.96	1.25
First >60 mins & second ≤45 mins	0.82	0.99	1.11
First ≤60 mins & second ≤45 mins	0.70[3]	0.88	1.53
**First >60 mins & second >60 mins**		**Reference**	
First ≤60 mins & second >60 mins	0.92	0.99	1.11
First >60 mins & second ≤60 mins	1.08	1.00	1.0
First ≤60 mins & second ≤60 mins	0.78[3]	0.90	1.46

[1]Effect size in odds ratio. [2]Effect size converted to relative risk. [3]Statistically Significant Association

© 2025 Gwarzo I et al. *JAMA Pediatrics*.
