# Supplementary material for: Opioid Timeliness in the Emergency Department and Hospitalizations for Acute Sickle Cell Pain
Source: JAMA Pediatr. 2025 Sep 2;179(11):1194–202. doi: 10.1001/jamapediatrics.2025.2967 (PMC12406144; doi:10.1001/jamapediatrics.2025.2967)
Supplement: Supplement 2. — Data Sharing Statement. [file jamapediatr-e252967-s002.pdf]

Data Sharing Statement

Gwarzo. Opioid Timeliness in the Emergency Department and Hospitalizations for Acute Sickle Cell Pain. *JAMA Pediatr*. Published September 02, 2025.
doi:10.1001/jamapediatrics.2025.2967

Data
Data available: No

Additional Information
Explanation for why data not available: The data supporting this study's findings are available from the Pediatric Emergency Care Applied Research Network (PECARN) Registry, housed at the University of Utah. Restrictions apply to the availability of these data, which are used under license for this study. Data are available from the PECARN/University of Utah when they become available as a public-use dataset, with the permission of the PECARN Executive Committee.
